# Integrating spatial transcriptomics and single‐cell RNA‐seq dissects immune microenvironment in fatty liver regeneration

**DOI:** 10.1002/ctm2.70365

**Published:** 2025-06-13

**Authors:** Chenhao Xu, Renyi Su, Yisu Song, Wenzhi Shu, Mengfan Yang, Zhe Yang, Xiao Xu, Xuyong Wei

**Affiliations:** ^1^ Department of Hepatobiliary and Pancreatic Surgery Hangzhou First People's Hospital, Zhejiang University School of Medicine Hangzhou China; ^2^ Institute of Translational Medicine, Zhejiang University School of Medicine Hangzhou China; ^3^ Department of Organ Transplantation Qilu Hospital of Shandong University Jinan China; ^4^ Department of Hepatobiliary and Pancreatic Surgery Department of Liver Transplantation Shulan (Hangzhou) Hospital, Zhejiang Shuren University School of Medicine Hangzhou China; ^5^ School of Clinical Medicine, Hangzhou Medical College Hangzhou China; ^6^ NHC Key Laboratory of Combined Multi‐organ Transplantation Hangzhou China; ^7^ Key Laboratory of Integrated Oncology and Intelligent Medicine of Zhejiang Province Hangzhou China

1

Dear Editor,

Liver zonal regeneration in healthy states involves a dynamic interplay between parenchymal and nonparenchymal cells, whereas fatty liver chronicity disrupts immune‐niche coordination, altering intercellular crosstalk.^1^, ^2^ While single‐cell technologies resolve cellular heterogeneity, they often overlook spatial regulation of cellular functions. We performed PHx (partial hepatectomy) on healthy mice and mice on a high‐fat diet, and sampling was performed on postoperative days 0, 2, 4, and 6. Our study integrates spatial transcriptomics with single‐cell profiling, bulk RNA‐seq, and smFISH to construct a spatiotemporal atlas of liver regeneration post‐PHx.

We constructed single‐cell maps (105 442 cells after quality control) and spatial maps (25 995 points) depicting the dynamics of liver regeneration after hepatectomy in normal and fatty livers (Figure 1A,B). Batch‐effect‐free integration (Figure S1A–C) confirmed impaired ecological niche coordination in the fatty microenvironment. Clustering identified 14 major cell types (annotated by literature calibration markers; Figure 1C; Table S1), in which Kupffer/endothelial cells—key regenerative mediators—were reduced in the fatty liver state and parenchymal cells (hepatocytes/BECs) were diminished (Figure 1D; Figure S1B).^3^, ^4^ Spatial validation confirmed lipid‐laden in the fatty model (Figure 1E). The temporal analysis highlighted a different immune response: fatty livers exhibited an early neutrophil/monocyte surge at day 2 (Figure S1D), in contrast to the 48 h regeneration peak in normal livers.^5^ These data establish a spatiotemporal map that identifies fatty liver‐specific defects in parenchymal‐immune crosstalk and delayed regenerative activation.

**FIGURE 1:**
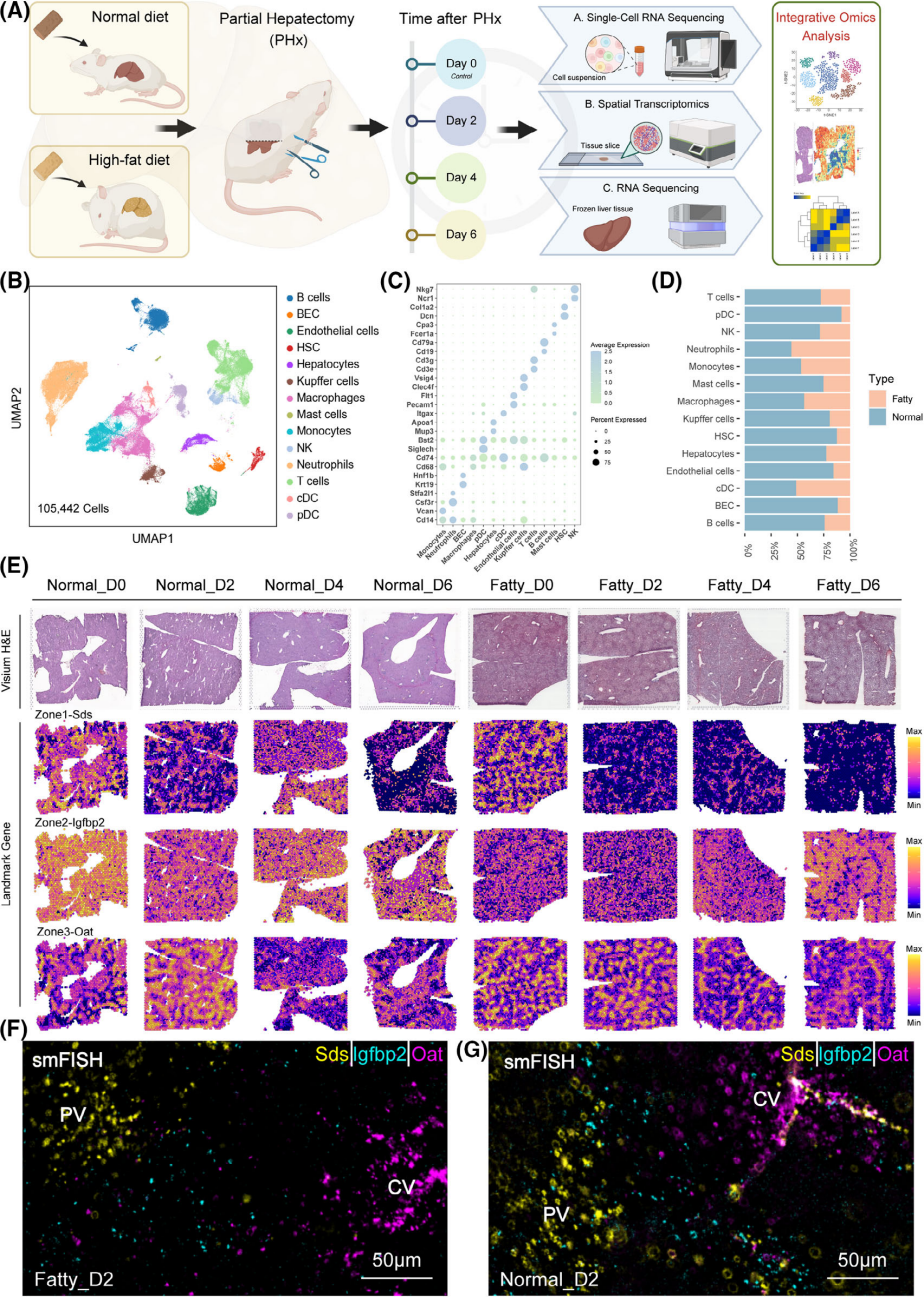
Spatiotemporal multi‐omic profiling of liver regeneration in normal and fatty liver after PHx. (A) Schematic overview of the strategy integrating scRNA‐seq, spatial transcriptome and bulk RNA sequencing in this study. (B) UMAP of 14 subtypes using scRNA‐seq data across all mouse samples after quality control (*n* = 105 442 cells). (C) Dot plot of representative markers for annotation of major types using single‐cell data (Also see Table S1). (D) Cell composition of major types in normal and fatty liver after PHx. (E) Spatiotemporal characterization of mouse liver regeneration for raw H&E image (upper panel) and representative zonal genes (lower panel). (F, G) Spatial validation of three zonal genes stained with Sds (Zone 1, yellow), Igfbp2 (Zone 2, cyan), and Oat (Zone 3, magenta) by smFISH at normal and fatty status. Scale bars, 50 µm. BEC, biliary epithelial cell; CV, central vein; HSC, hepatic stellate cell; NK, natural killer cell; DC, dendritic cell; PHx, partial hepatectomy; PV, portal vein.

Meanwhile, spatial mapping validated the classical 1, 2, and 3 zonation markers (Sds/Igfbp2/Oat; Figure 1E–G) and identified 7 molecular niches after PHx (Figure S1E–H).^6^, ^7^, ^8^ To further characterize the features of each molecular niche, we performed differential analysis to identify the representative markers (Table S2). Niche 2 and niche 1/6 expressed Cyp2f2/Sds and Cyp2e1/Oat, respectively, whereas midlobular niche 3/4 was enriched in Igfbp2.^6^ The molecular niches might be representative of the spatial structure (Figure 2A,C). Spatial projection confirmed niche‐structure alignment: the zonal structure remains clear and significant in different disease states and at different points in time (Figure 2B). Pathway analysis showed attenuated Wnt activation (Figure 2E,F) and similarly attenuated TGF‐β inhibition (Figure S2A,B) in fatty liver compared with normal liver.^9^, ^10^ Single‐cell profiling showed that regeneration of hepatocytes in the fatty liver was delayed—Wnt signalling and proliferative activity peaked on day 4, whereas in the normal group, it peaked on day 2 (Figure 2D,G; Figure S2C,D). This temporal variation was also confirmed by bulk RNA‐seq and western blot results of cell cycle markers such as Pcna (Figure 2H,I). These data establish that fatty liver retains regenerative capacity but with impaired spatiotemporal coordination, evidenced by disorganized niche architecture, desynchronized Wnt/TGF‐β signalling, and delayed proliferation kinetics.

**FIGURE 2:**
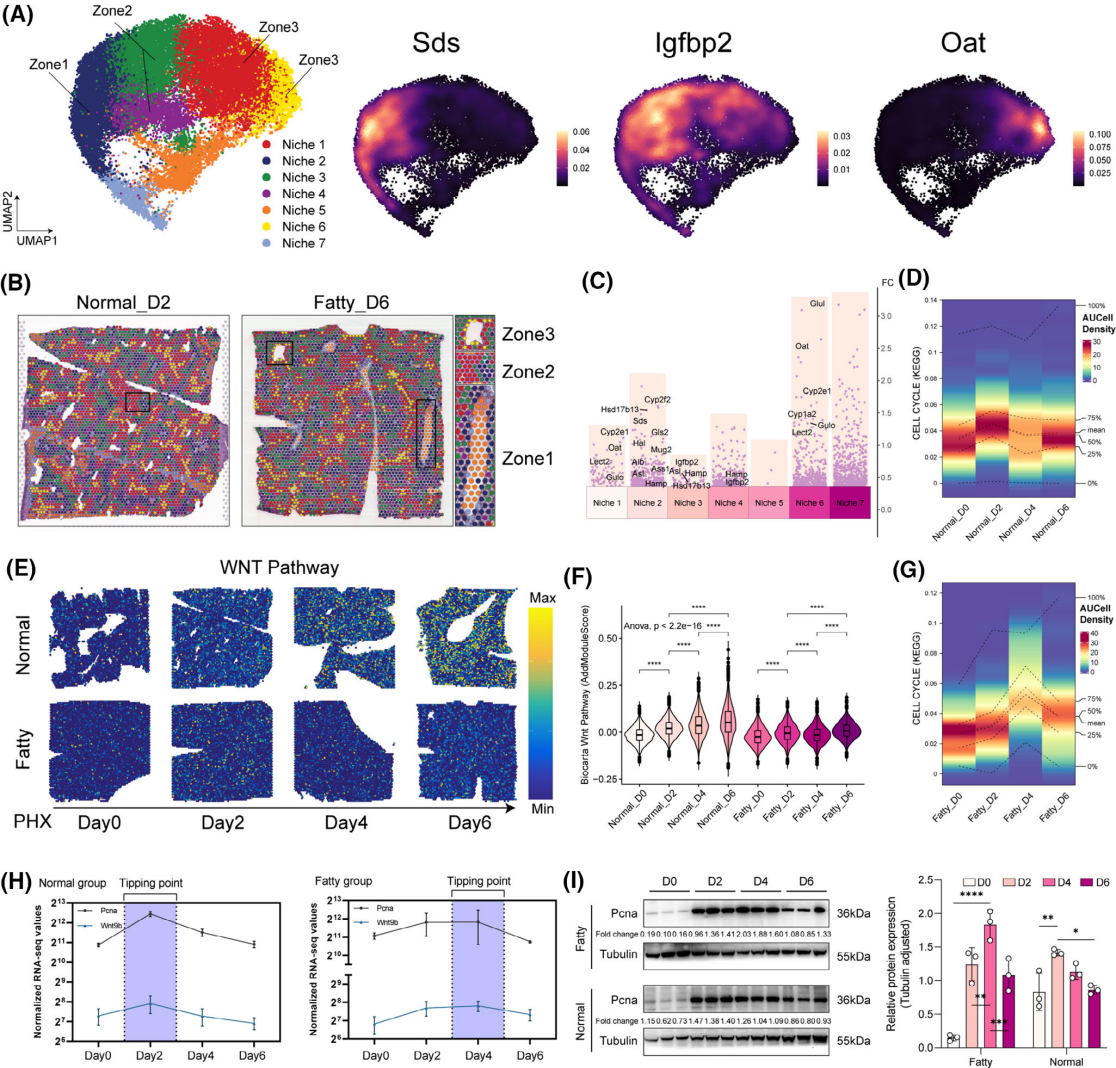
Liver regeneration is delayed in fatty liver after PHx. (A) UMAP of molecular niches integrating spots across all slides (left panel). Representative zonal markers of Zone 1 (Sds), Zone 2 (Igfbp2) and Zone 3 (Oat; right panel). (B) Representative slides mapping molecular niches onto raw H&E images. The right black blanket in Normal_D2 slide showed Zone 2 region. The middle and right black blankets in Fatty_D6 slide indicated Zone 3 and Zone 1 regions, separately. (C) Volcano plot of upregulated markers in each molecular niche labelled with previously reported zonation markers (calculated by FDR using a two‐sided Wilcoxon rank‐sum test). The *y*‐axis is the fold change of genes in each molecular niche. The activity of the Cell cycle was shown in AUCell density in normal (D) and fatty (G) status at different time points after PHx. (E) General activity of Wnt pathway of spots across all slides at different time points and status and (F) calculated by AddModuleScore method in Seurat ((*p*‐value, two‐sided Wilcoxon rank‐sum test). (H) Dynamic expression of two representative proliferation markers (Pcna and Wnt9b) by RNA‐seq with normalized value. (I) Immunoblots of each group of Pcna at different time points in normal and fatty states, and their quantitative statistical plots. Tubulin serves as a loading control. *n* = 3 independent biological replicates. The data are presented as the means ± SD (*n* = 3). *, *p *< .05; **, *p *< .01; ***, *p *< .001;****, *p *< .0001. Scale bars, 50 µm. PHx, partial hepatectomy; UMAP, uniform manifold approximation and projection.

We integrated single‐cell data from hepatocytes and cholangiocytes and noted a specific subpopulation, Vim^+^Cd44^+^ interface hepatocytes (Figure 3A). In the fatty state, interface hepatocytes had an enhanced proliferative capacity compared with other hepatocytes. However, Hippo/Wnt signalling remained strong in the normal state (Figure 3B,C). Spatial mapping with smFISH showed significant enrichment of Vim^+^Cd44^+^ interface hepatocytes in fatty liver at the peak of regeneration (day 4, Figures 3D, S3A–D). These interface cells showed regional redistribution: at the peak of normal regeneration (day 2) they were enriched in zone 3, whereas at the peak of fatty state regeneration (day 4) they were enriched in zone 1 (Figure 3E). Fatty liver is less regenerative, and Vim^+^Cd44^+^ interface hepatocytes may be a compensatory response to stress, acting as a transient intermediate to compensate for impaired regenerative signalling.

**FIGURE 3:**
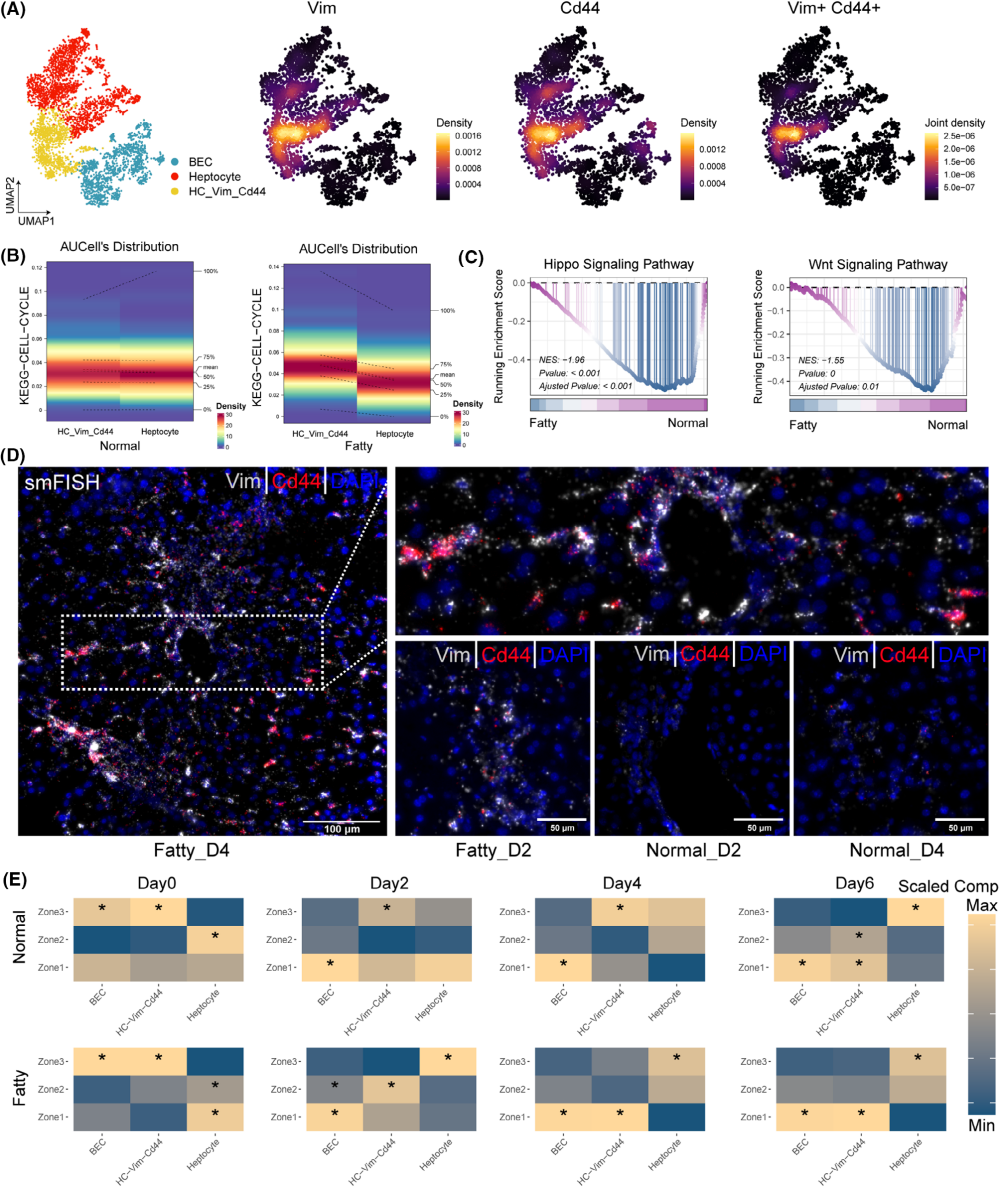
Interface hepatocyte's initial oncofetal genes reprogram fatty liver regeneration. (A) UMAP visualization of hepatocytes and BECs identifies Cd44^+^Vim^+^ interface cells, coloured by cell type (left panel) and expression intensity (right panel). (B) Cell proliferation ability of interface cell and hepatocytes among normal (left panel) and fatty status (right panel). The AUCell method is used to calculate the KEGG cell cycle score. (C) Representative pathways of liver regeneration among normal and fatty status profiling by GSEA method. (D) smFISH of interface cell. Cell nuclei are stained with DAPI (blue) and early hematopoietic markers Cd44 (red), and Vim (grey). Scale bars are indicated in the figures. (E) Scaled median compositions of major cell types within each Zonation. Asterisks indicate the increased composition of a cell type in a niche compared with other niches (one‐sided Wilcoxon rank sum test, adjusted *p* <.05). BEC, biliary epithelial cell; GSEA, gene set enrichment analysis; PHx, partial hepatectomy; UMAP, uniform manifold approximation and projection.

Single‐cell analysis identified 12 T cell subsets and characterized the cytotoxic, immunosuppressive, and inflammatory capacities of the T subpopulations (Figure 4A–C), with Foxp3^+^Ctla4^+^CD4^+^ Tregs and Ccl5^+^CD8^+^ T cells exhibiting dynamic regulation during regeneration. In normal liver, both subsets decreased at day 2 (pro‐regeneration phase) before rebounding post‐repair, while fatty liver displayed dysregulation with early CD8^+^ T cell accumulation and insufficient Treg expansion (Figure 4D). Compared with the other timepoint, the Cd4^+^ Tregs specifically re‐located itself to Zone 3 to conduct the immunosuppressive function, while Ccl5^+^ Cd8^+^ T seemed to be relatively sparse over Zone 2 and Zone 3 to harness the regeneration at both regenerations’ turning point (Day 2 in normal status, Day 4 in fatty status, Figure 4E, Figure S3E–G).

**FIGURE 4:**
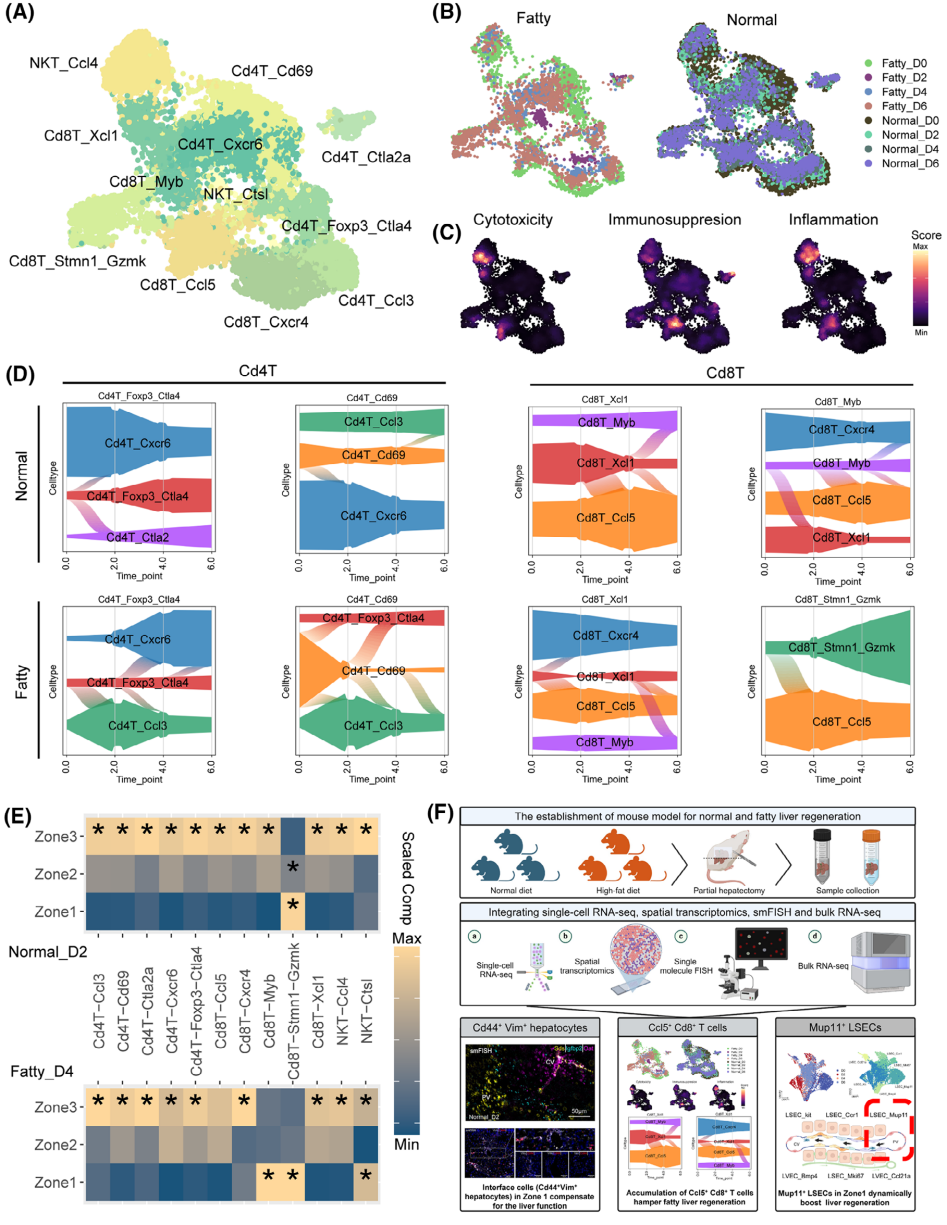
Excessive adaptive immune activation and inflammatory response in microenvironment hamper regeneration in fatty liver after PHx. UMAP visualization of T cell, coloured by subtypes (A), groups and timepoints (B), and phenotype (C). The phenotypes are calculated by the AddModuleScore method in Seurat and projected onto UMAP. (D) Shifts of T cells fate among normal (upper panel) and fatty status (lower panel) by CellRank algorithm. (E) Scaled median compositions of T cell subgroups within each Zonation (also see Figure S3E). Asterisks indicate the increased composition of a cell type in a niche compared with other niches (one‐sided Wilcoxon rank sum test, adjusted *p* < .05). (F) Summary: Regenerative capacity is impaired in fatty liver and hepatocytes may require an intermediate state (termed Cd44+Vim+ interface cells) to maintain function. CD4 Treg insufficiency and day 2 Ccl5+ CD8 T cell accumulation drive immune dysregulation, disrupting fatty liver regeneration. Nomination of Mup11+ LSEC located in Zone 1 potentially promotes liver regeneration. PHx, partial hepatectomy; UMAP, uniform manifold approximation and projection.

Moreover, the single‐cell analysis identified six endothelial subtypes (Figure S4A,B) with zonal specialization. The endothelial cells were well located with Kit^+^ LSCEs and Bmp4^+^ LVECs located in Zone 3, Mup11^+^ LSECs and Ccl21a^+^ LVECs located in Zone 1, while Ccr1^+^ LSECs and Mki67^+^ LSEC located between Zone 1 and Zone 3 (Figure S4C,D). Zone 3 endothelial cells were enhanced in regenerative pathways such as PI3K‐Akt and Hippo signalling pathways (Figure S4E). We noticed the immune suppressive function of Mup11^+^ LSCEs showed a similar trend to liver regeneration ability, indicating this LSCE might be another essential endothelial cell in liver regeneration (Figure S4F).

Myeloid profiling identified 13 subsets (Figure S5A,B). We focused on a subgroup termed Ace^+^ monocytes (Figure S5C). This subgroup is more abundant on day 2 in normal status and on day 4 in fatty status, suggesting that it may have some contribution to liver regeneration (Figure S5D). smFISH validated the results (Figure S5F–I). We explore the function of Ace^+^ monocytes showing this subtype is mainly involved in injury repair functions including focal adhesion, platelet activation, and phagocytosis to remove dead cells and avoid immune response (Figure S5E).

In summary, the fatty liver shows a delay in regeneration compared with normal liver after PHx. The intermediate fetal state (Vim^+^Cd44^+^ interface hepatocytes), however, may be an important source of fatty liver regeneration. The microenvironment of fatty liver was reshaped to hamper the liver regeneration with excessive immune function by insufficient Cd4^+^ Tregs and enhanced injury response by early accumulated Ccl5^+^ Cd8^+^ T at day 2. We also identified several subgroups that potentially promote liver regeneration such as Mup11^+^ LSEC located in Zone 1 and Ace^+^ monocyte more enriched at liver regeneration turning point (Figure 4F). Spatial positioning dictates niche‐specific cellular functions across parenchymal and nonparenchymal compartments. Our spatiotemporal mapping of fatty liver microenvironments reveals therapeutic targets for cell‐type or location‐based regenerative strategies.

## AUTHOR CONTRIBUTIONS

Chenhao Xu and Renyi Su: Conceptualization, methodology, data curation, formal analysis, software, resources, project administration, visualization, and writing—original draft. Yisu Song: Writing—review & editing. Wenzhi Shu: Data Curation and writing—review & editing. Mengfan Yang: Data curation. Zhe Yang: Conceptualization and supervision. Xiao Xu: Conceptualization, writing—review & editing, funding acquisition, and supervision. Xuyong Wei: Conceptualization and funding acquisition. All authors have approved the submission of the manuscript.

## CONFLICT OF INTEREST STATEMENT

The authors declare no conflict of interest.

## ETHICS STATEMENT

There are no human studies in this manuscript. Animal studies were reviewed and approved by the Institutional Animal Care and Use Committee, ZJCLA (no. ZJCLA‐IACUC‐20220077) to confirm humane care on the use of vertebrate animals.

## Supporting information




**Supporting File 1**: ctm270365‐sup‐0001‐SuppMat.docx

**Supporting File 2**: ctm270365‐sup‐0002‐TableS1.xlsx

**Supporting File 3**: ctm270365‐sup‐0003‐TableS2.xlsx

**Supporting File 4**: ctm270365‐sup‐0004‐TableS3.xlsx

## Data Availability

The datasets used and/or analyzed during the current study are available from the corresponding author upon reasonable request.
