# Supplementary material for: Integrating spatial transcriptomics and single‐cell RNA‐seq dissects immune microenvironment in fatty liver regeneration
Source: Clin Transl Med. 2025 Jun 13;15(6):e70365. doi: 10.1002/ctm2.70365 (PMC12166122; doi:10.1002/ctm2.70365)
Supplement: Supplementary file 1 — Supporting File 1: ctm270365‐sup‐0001‐SuppMat.docx [file CTM2-15-e70365-s003.docx]

**Integrating spatial transcriptomics and single-cell RNA-seq dissects immune microenvironment in fatty liver regeneration**

**Chenhao Xu^1, 3, #^, Renyi Su^1, 3, #^, Yishu Song^1, 3, #^, Wenzhi Shu^1, 3^, Mengfan Yang^5^, Zhe Yang^6, *^, Xiao Xu^2, 3, 7, *^, Xuyong Wei^1, 4, *^**

^1^Department of Hepatobiliary and Pancreatic Surgery, Hangzhou First People's Hospital, Zhejiang University School of Medicine, Hangzhou, China.

^2^School of Clinical Medicine, Hangzhou Medical College, Hangzhou, China.

^3^Institute of Translational Medicine, Zhejiang University School of Medicine, Hangzhou, China

^4^Key Laboratory of Integrated Oncology and Intelligent Medicine of Zhejiang Province, Hangzhou, China.

^5^Department of Organ Transplantation, Qilu Hospital of Shandong University, Jinan, China.

^6^Department of Hepatobiliary and Pancreatic Surgery, Department of Liver Transplantation, Shulan (Hangzhou) Hospital, Zhejiang Shuren University School of Medicine, Hangzhou, China.

^7^NHC Key Laboratory of Combined Multi-organ Transplantation, Hangzhou, China

**^*^ Correspondence**

Dr. Zhe Yang, Department of Hepatobiliary and Pancreatic Surgery, Department of Liver Transplantation, Shulan (Hangzhou) Hospital, Zhejiang Shuren University School of Medicine, Hangzhou, China.

Prof. Xiao Xu, School of Clinical Medicine, Hangzhou Medical College, Hangzhou, China.

Dr. Xuyong Wei, Department of Hepatobiliary and Pancreatic Surgery, Hangzhou First People's Hospital, Hangzhou, China. E-mail addresses: 1315009@zju.edu.cn

**^#^ These authors contributed equally: Chenhao Xu, Renyi Su, and Yishu Song.**

**Supplementary figure legends**

**
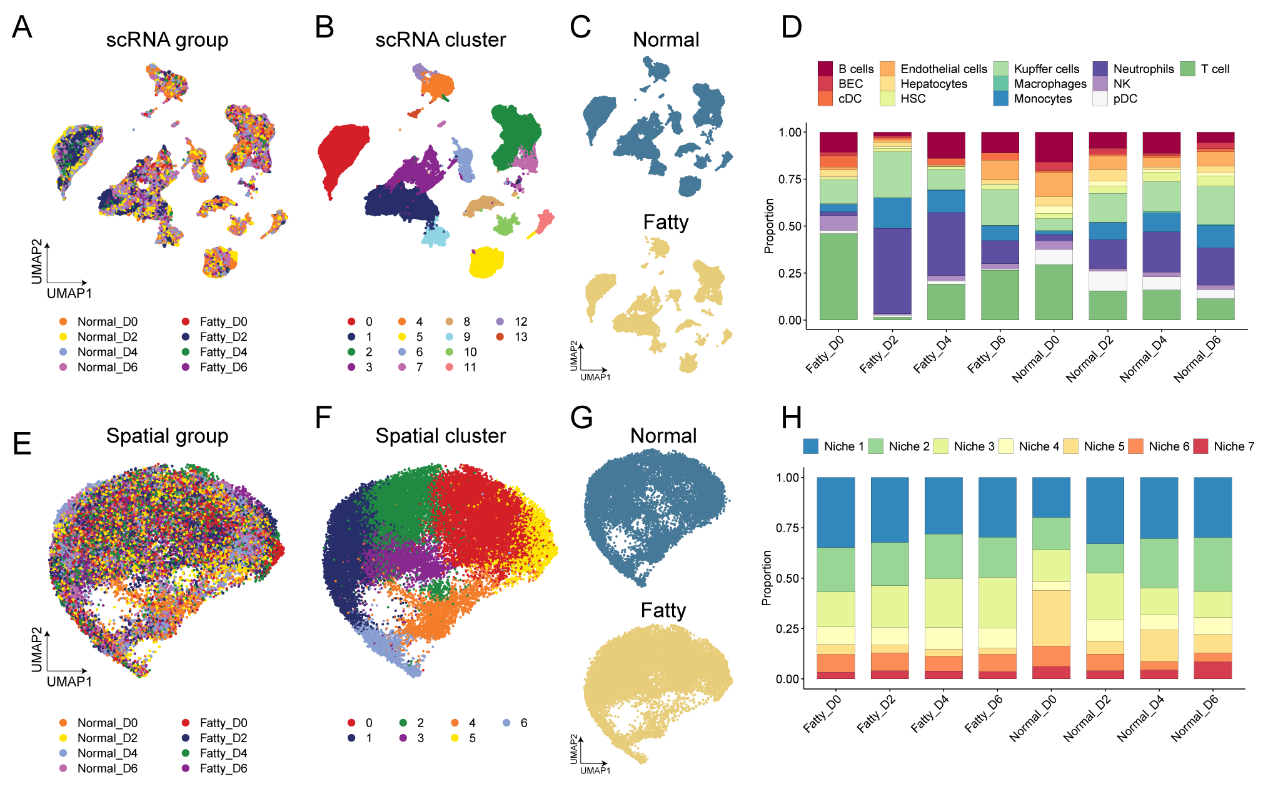
**

**Fig. S1** Quality control of single-cell data and spatial transcriptome in mouse liver regeneration. UMAP of scRNA (A) and spatial data (E) facet by time points in normal and fatty conditions. UMAP of scRNA (B) and spatial data (F) by Louvain clusters. UMAP of scRNA (C) and spatial data (G) by sample type. Dynamics in the composition of tailor annotated cell subtypes (D) and molecular niches (H) across time span after PHx. PHx, partial hepatectomy; UMAP, uniform manifold approximation and projection.


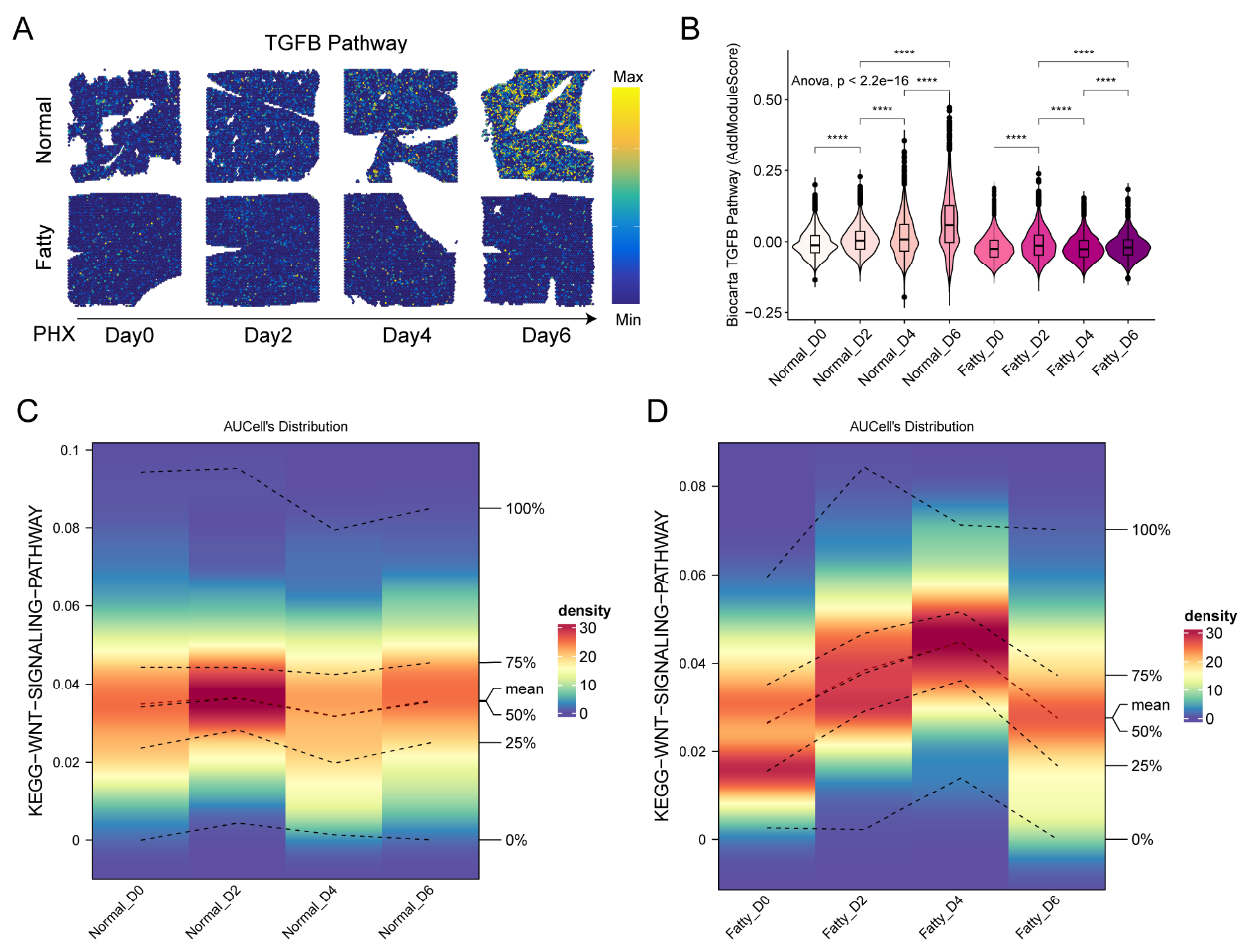


**Fig. S2** Spatial and single cell analysis of signaling pathway. (A) General activity of TGF-beta pathway of spots across all slides at different time points and status and (B) calculated by AddModuleScore method in Seurat ((P-value, two-sided Wilcoxon rank-sum test) related to Fig. 2. (C, D) The activity of Wnt signaling pathway is shown in AUCell density in normal (C) and fatty (D) status at different time point after PHx. Related to Fig. 3.


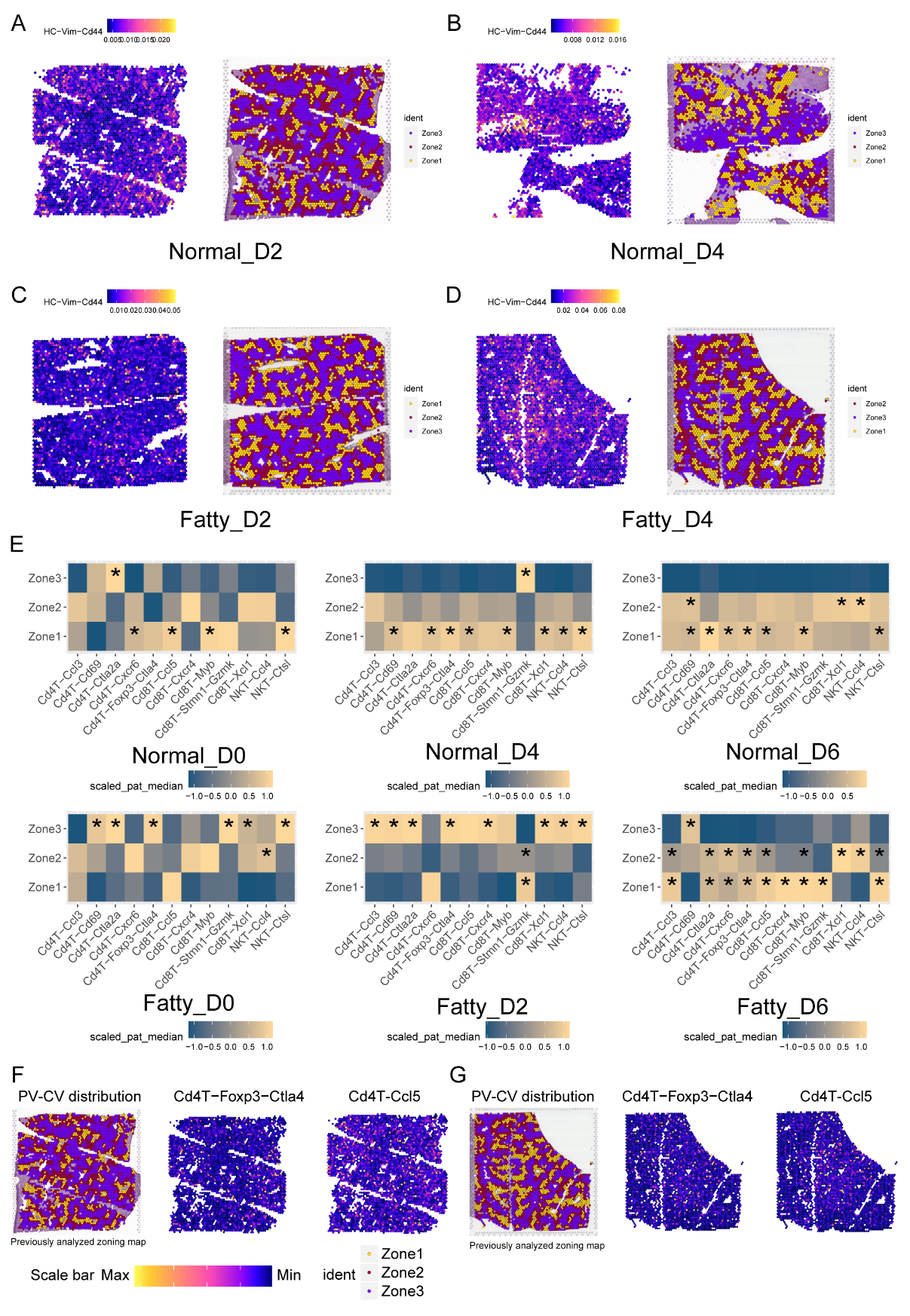


**Fig. S3** Spatial distribution of Vim^+^Cd44^+^ interface cells among normal (A, B) and fatty status (C, D) visualized in situ H&E images. (E) Scaled median compositions of T cell subgroups within each Zonation (related to Fig. 4). (F and G) Spatial distribution of Cd4 Tregs and Ccl5^+^ Cd4 T cells among normal and fatty status visualized in situ H&E images. Asterisks indicate increased composition of a cell type in a niche compared with other niches (one-sided Wilcoxon rank sum test, adjusted P < 0.05).


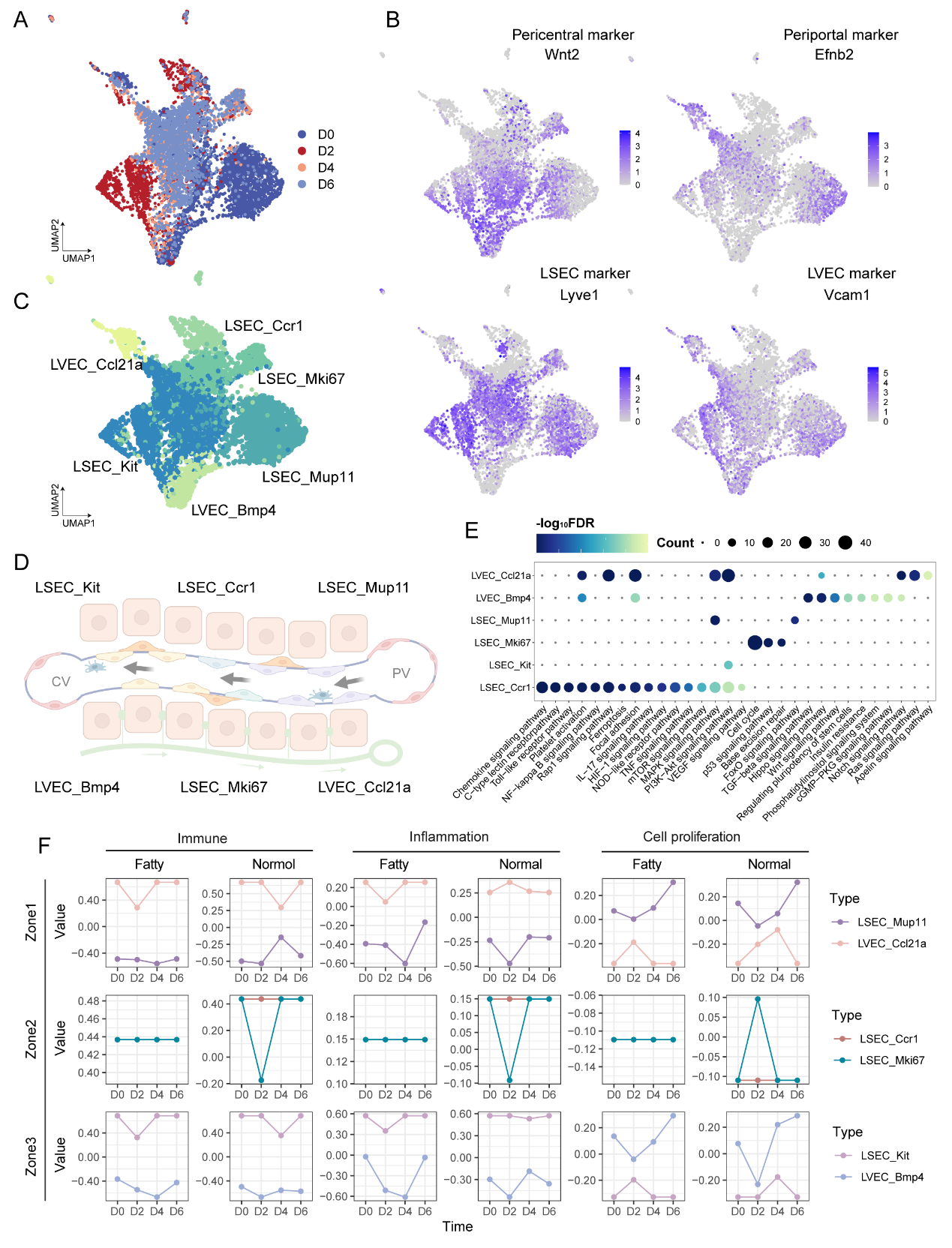


**Fig. S4** Dynamics of endothelial cell subgroups exhibit distinct zonal expression programs. (A) UMAP visualization of endothelial cells, colored by time point. (B & C) UMAP visualization of endothelial cells, colored by the expression level of the pericentral Wnt2 (top left), periportal Efnb2 (top right), sinusoidal endothelial cell marker Lyve1 (bottom left), and vascular endothelial cell marker Vcam1 (bottom right). (D) Schematic figure of endothelial cell subgroups among the PV-CV axis. (E) Pathway enrichment of endothelial cell subgroups. (F) Temporal dynamics of endothelial cells profiling by immune suppression score, inflammation score, and cell proliferation score. CV, central vein; PV, portal vein; UMAP, uniform manifold approximation and projection.


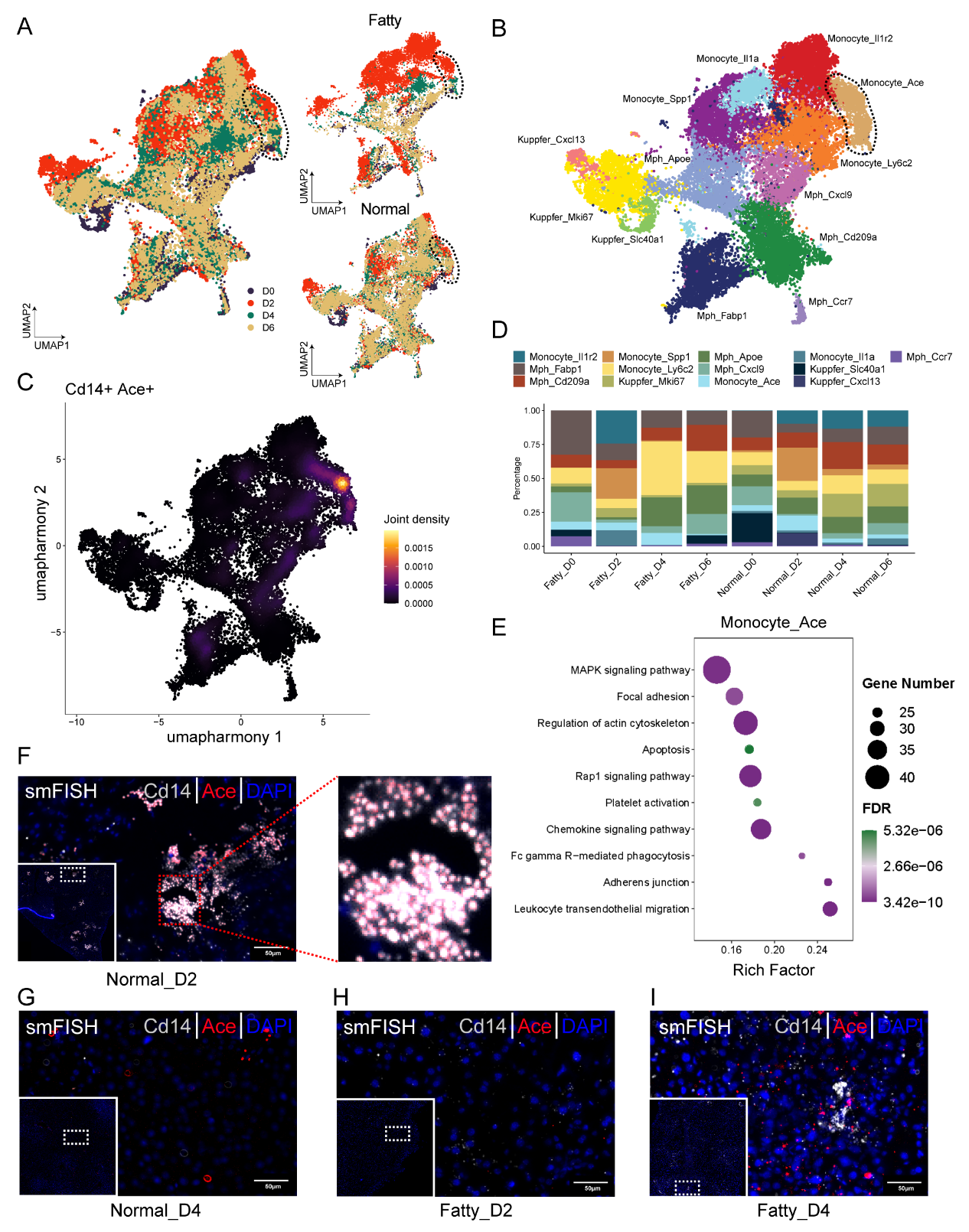


**Fig. S5** Spatiotemporal analysis of myeloid subgroups in mouse liver regeneration. (A) UMAP visualization of myeloid cells, colored by groups and time points (A) and subtypes (B). (C) UMAP visualization of Cd14^+^Ace^+^ monocyte subgroup. (D) The fractions of different myeloid cell subtypes at each time point. (E) Functional enrichment of Cd14^+^Ace^+^ monocyte. (F) smFISH of scan (top) and zoomed-in insets (upper right) of Cd14^+^Ace^+^ monocyte subgroup at day 2 in normal group. smFISH of Cd14^+^Ace^+^ monocyte subgroup at normal day 4 (G), fatty day 2 (H) and fatty day 4 (I). Nuclei are stained with DAPI (blue) and Cd14 and Acc are stained with gray and red, respectively. Scale bars, 50 μm. UMAP, uniform manifold approximation and projection.

**Materials and methods**

**Experimental model and subject details (including origin, feeding plan, and surgery details)**

All experimental procedures were approved by the Institutional Animal Care and Use Committee, ZJCLA (No.ZJCLA-IACUC-20220077). Adult male C57BL/6J mice (Gempharmatech Co., Ltd) aged 6-8 weeks were housed in a certificated SPF grade facility on a regular 12-hour light-dark cycle. Mice in normal group were fed with free access standard chow food, while fatty group was maintained on a high-fat diet (HFD, 60% calories from fat, SDS, 824054) for a period of 2 months. All mice had free access to drinking water. The 70% PHx procedure was performed on mice according to the previously reported protocol ^1, 2^. Mice were sacrificed at 0, 48, 96, or 144 hours after PHx to obtain liver tissue (16 mice) for paired scRNA-seq or spatial transcriptome.

**Tissue dissociation, cell isolation**

The liver tissue dissociation and cell isolation protocols were developed based on previously published methods ^3, 4^. We strictly followed standard procedures for single cell isolation of liver. Pre-cooled PBS was perfused through the portal vein until it faded from maroon to white and the effluent was clarified to minimize the effect of circulating blood. Liver samples were obtained under aseptic conditions, then rinsed 3 times with PBS and cut into small pieces with a scalpel (the sampling site was uniformly the right middle lobe in all groups).

The samples were digested in an incubating shaker at 37 °C and 200 RPM for 30-40 min. The enzymatic reaction was terminated by adding 1:1 ratio of 20% FBS to 80% PBS and the digest was filtered through a staged 100 μm and 40 μm filter. The dissociated cells were centrifugated at 300g for 5 min to collect cell fractions, followed by removing supernatant and incubating for 3 min in red blood cell lysis solution (Miltenyi Biotec, 130-094-183). MACS Dead Cell Removal Kit (Miltenyl Biotec, 130-109-398) was used following the manufacturer’s guidelines to remove dead cells and yield a clean cell pellet for the following analysis.

**Droplet-based scRNA-seq on 10x genomics platform**

The clean pelleted cells were resuspended, loaded into Chromium microfluidic chips (Chromium Next GEM Chip G Single Cell Kit) with 3’ chemistry, and barcoded with a 10× Chromium Controller (10X Genomics). The single-cell sequencing libraries were constructed using Chromium Next GEM Single Cell 3ʹ Library Kit v3.1& Dynabeads™ MyOne™ SILANE and sequenced with Illumina NovaSeq 6000 following the manufacturer’s instructions.

**Spatial gene expression assay on 10x Visium platform**

The spatial transcriptome was conducted using the Visium Spatial Gene Expression platform (10X Genomics). The protocol was consistent with the best practice of previously published work using the 10X Visium platform ^5^. Briefly, fresh mice liver tissues were embedded in OCT (Tissue-Tek) and diced into 10 µm sections using a cryostat (ThermoFisher Scientific). The sections were placed on the Visium Spatial Tissue Optimization Slide (Visium, 10X Genomics, PN-1000193) and the tissue optimal permeabilization time was determined using the Visium Spatial Tissue Optimization Slide & Reagents Kit with 18 min of digestion. The brightfield histological images were taken with the Leica Aperio Versa 200 scanner. The library's construction and sequencing were conducted according to the Visium user guide. A standard 300 pM sample loading to construct library followed by sequencing on NovaSeq 6000 System (Illumina platform) as recommended by 10X Genomics.

**RNA sequencing analysis**

Whole RNA sequencing experiments were conducted by LC-Bio (Hangzhou, China).

Briefly, to enhance the reliability, three biological replicates were used in the following eight groups with a total of 24 mice: Fatty_D0, Fatty_D2, Fatty_D4, Fatty_D6, Normal_D0, Normal_D2, Normal_D4 and Normal_D6. Freeze-snap specimens were used to extract total RNAs by the TRIzol method and labeled with sequence tags by UMI technology.

**Hematoxylin and eosin (H&E) staining and histology analysis**

H&E staining was conducted as previously described ^6^. The paraffin-embedded mouse tissues were sectioned at 5 μm thickness using a Leica CM3050S cryomicrotome. The slides were deparaffinized and re-hydrated in a sequential series of alcohols (100%, 95%, 80%, 75%). After washing with distilled water, the rehydrated sections were incubated in hematoxylin and then 1% acid alcohol for 30 s. Afterward, the sections were counterstained with eosin and dehydrated in alcohols and xylene before mounting with Cytoseal-60 (Stephens Scientific). The images were taken with a Leica DM 1000 LED microscope and a Leica MC120HD microscope camera using LAS V4.4 software (Leica).

**Quantification and statistical analyses**

**scRNA-seq data processing**

In this study, we used Cell Ranger Analysis Pipeline (version 6.0.2) to generate sequencing libraries from single-cell transcriptomes with default options. The alignment of sequences was based on the FASTQ files, a reference genome file (mouse:mm10), and a transcriptome annotation file (mouse: GENCODE vM23/Ensembl 98).

The analysis of scRNA data refers to the best practice of recently published works ^7, 8^. Briefly, the Seurat R package (version 4.3.0) was used for the whole pipeline including data preprocess filtering, normalization, dimensionality reduction, clustering, and visualization. Firstly, we performed quality control processing on individual scRNA-seq data based on the following parameters. (1) Cells with at least 200 genes and 500 UMI, (2) with less than 30% of mitochondrial gene expression, (3) genes expressed at least in five cells, (4) doublets were removed using the DoubletFinder package (version 2.0.3) with default parameters were excluded for the analysis. To avoid unexpected noise and expression affected by dissociation in the scRNA-seq study, we excluded the gene set provided by Xue et al before data integration ^9^. The SCTransform function in Seurat was performed on all samples individually to normalize data, find high-variable genes, and then scale the data. The integrated atlas was constructed using principal component analysis (PCA) with the top 40 principal components based on the shared top 3,000 most variable genes among all samples. Since the batch exists among different time points, the integration was conducted using a harmony algorithm to correct PCA with each sample as an individual covariate. The same 40 principal components were used to build a Euclidean distance-based shared nearest neighbor graph using the FindNeighbors function in Seurat. Cells were clustered using the Louvain-based FindClusters function with the optimal resolution selected by the cluster R package (version 0.5.0). The visualization of dimensionality reduction on uniform manifold approximation and projection (UMAP) algorithm with RunUMAP function using first 40 principal components. Cluster markers were identified using the FindAllMarkers function with default Wilcoxon tests implemented in Seurat (Top 50 genes with adjusted P < 0.05 using Bonferroni correction). The well-known marker genes were assigned to annotate major cell types. Details of representative markers for liver regeneration atlas construction were summarized in **Table S1**. Next, the subpopulations of each major cluster were re-analyzed generally the same as the aforementioned pipeline, but differed in hvgs with 2000 hvgs for myeloid cells and T cells, 1000 hvgs for endothelial cells and hepatocytes.

The cell type composition was calculated as the ratio of a given cell type dividing by all cells in individual samples at each time point. Enrichment analysis of differentially expressed genes was conducted using the clusterProfiler R package (version 4.7.1.003) with KEGG pathway databases.

**Single-slide spatial transcriptomics processing**

The SpaceRanger Analysis Pipeline (version 1.3.2) was used to pre-process the sequencing data with default settings. The general analysis principle of spatial transcriptomics referred to previously published work ^7^. Quality controls were initially performed for each slide with the following parameters using the Seurat package (version 4.3.0). (1) Spots with less than 300 measured genes per spot and 500 UMI counts per spot were removed. (2) Ribosomal, mitochondrial, and hemoglobin genes were excluded from the following analysis. The spatial hvgs were identified using with SPARKX algorithm (version 1.1.1) with adjusted P < 0.05 using Bonferroni correction. Each sample was normalized with SCTransform and comparative log1P normalization and scaled matrices. The dimensionality reduction, clustering, and UMAP visualization method and parameter settings were the same as the scRNA data processing procedure. The overrepresented analysis of spatially variable genes was carried out using hypergeometric tests (adjusted P value < 0.05) on the gene set curated from MSigDB (msigdb_v2022.1.Mm_files).

**Niche identified from spatial transcriptomics data**

To characterize the similar spatial molecular niche features among different samples, we referred to the method used in single-cell atlas construction. Briefly, each single slide was processed individually to curate the hvgs, and the SCTransform normalization method was carried on the top 3000 hvgs among all samples followed by the same harmony integration method, dimensionality reduction, clustering, and UMAP visualization.

**Liver lobule spatial analysis**

The construction of spatial coordinates of mouse liver using the 10X Genomics Visium platform was recently reported by Hildebrandt et al ^10^. The similar approach was adopted to construct liver lobule spatial coordinates. Briefly, we first used the same criteria to obscure markers of each molecular niche. Each molecular niches were compared to the rest using SCT-normalized expression among all spots in the same niche. Representative markers of each niche were defined as genes with log2 fold change (FC) > 0.25 and Benjamini–Hochberg adjusted P <0.05. By mapping the molecular niche markers to previously reported zonated landmark genes, lobes with significant zonated features were classified into zone1 to zone3 along porto-central axis. To compare compositional differences among different zones, pairwise comparisons were performed with the Wilcoxon test (adjusted P value <0.001 adjusted with Benjamini–Hochberg procedure) per sample type (Normal or fatty status) per time point (day 0, day 2, day 4, day 6).

**Spatial map of cell colocalization**

The state-of-art python-based Cell2location algorithm was adopted to deconvolute spatial data using pre-annotated major types and tailor types annotated single-cell data ^11^. The two annotated single-cell reference data were processed separately using regularized negative binomial regressions. Genes were filtered using the filter genes function in the cell2location python module with the parameters setting as gene expressed in at least 5 cells, genes expressed at least in 4% cells, mean gene expression over 1.2. Each sample was set as a batch corrected in regression model. The single-cell reference model was trained for 250 epochs to gain a relative plank ELBO loss. Next, the single slide was deconvoluted using hierarchical Bayesian models by implementing cell2location.models. Cell2location function. The number of cells per location was set as six according to the estimate by manually counting nuclei in 20 locations in the H&E image using 10x Loupe browser. The detection alpha was set as 20 for high variation in RNA detection while 200 for low variation in RNA detection based on quality control. The deconvoluted model was trained in 30000 epochs to gain a relative plank ELBO loss. The top 5% of cells were deemed as highly enriched cells within a spot and output the estimated absolute abundance for the following analysis. Cell composition was calculated as the ratio of absolute abundance of each cell dividing by the same cell type per slide.

**Trajectory analysis**

Cellular trajectories of T cells were reconstructed using the [RealTimeKernel](https://cellrank.readthedocs.io/en/stable/api/_autosummary/kernels/cellrank.kernels.RealTimeKernel.html#cellrank.kernels.RealTimeKernel) of the CellRank algorithm given our temporal experiment design^12^. Firstly, mascot was adopted to couple cells by setting up a TemporalProblem. Then, we used RealTimeKernel loading cellular couplings computed with moscot. Finally, we row-normalized the resulting cell-cell transition matrix (including all time points), constructed the Markov chain, and visualized the reconstructed dynamics by plotting the probability mass flow in time.

**Deconvolution of cell composition by BayesPrism**

Bulk RNA-seq data were deconvoluted by paired single-cell RNA data using the BayesPrism algorithm to faithfully recreate the cell proportion^13^. All the parameters were set as default. Here, we used the convoluted endothelial cell composition to evaluate the cellular molecular function by GSVA with spatiotemporal design referring to the gene sets previously validated^14^.

**Western blot**

The proteins extracted from mouse liver using a modiﬁed buffer were followed by immunoblotting using the Pcna (CST) at dilution 1:2000, Tubulin (CST) at dilution 1:1000, as described previously^15^.

**Single molecule ﬂuorescence in-situ hybridization (smFISH)**

Normal or fatty mice were sacriﬁced at two time points (48h and 96h). The specific targeting probes were designed by spatial FISH Ltd **(Table S3)**. The samples were fixed with 4% paraformaldehyde, and then covered with reaction chamber. After dehydration and denaturation of samples with methanol, the hybridization buffer with specific targeting probes was added to the chamber for incubation at 37°C overnight. Then samples were washed three times with PBST, followed by ligation of targeting probes in ligation mix at 25°C for 3 h. Next, samples were washed three times with PBST and subjected to rolling circle amplification by Phi29 DNA polymerase at 30°C overnight. Subsequently, the fluorescent detection probes in the hybridization buffer were applied to the samples. Finally, samples were dehydrated with an ethanol series and mounted with mounting medium. After capturing images, signal dots were decoded to interpret DNA spatial position information. All smFISH images were visualized with LAS X Core software (version 3.7.2).

**Statistical analysis**

Detailed quantitative and statistical analyses for each of the analyses are provided above. In general, Wilcoxon tests were used to evaluate statistical differences for continuous variables. If not other stated, p values were corrected in multi-comparison tests using the Benjamini–Hochberg method. The p < 0.05 is considered statistically significant in the study. Statistical analyses were performed using R (version 4.2.1) and Python (version 3.8.15) and GraphPad Prism (version 9.0.0-121).

**Supplementary description**

**Multi-omic map of liver regeneration at normal and fatty status**

To study the misty of immune microenvironment in fatty liver regeneration, a combined approach integrating single-cell RNA sequencing with spatial transcriptomics using 10X Genomics platform and validated by RNA-seq method was adopted to unravel the molecular dynamics at unprecedented spatial resolution. Previous studies showed regeneration of normal mouse liver peaks at 2 days (48 hours) following PHx. Since the fatty liver may potentially delay the liver regeneration process, we profiled mouse liver regeneration features in both normal status and fatty status with sequential time design at four time points following PHx (day 0, day 2, day 4 and day 6; Fig. 1A).

Paired 10 µm cryosections and directly adjacent liver tissues were curated for scRNA-seq (16 mice) and spatial transcriptome study (8 mice) and bulk RNA-seq (24 mice). Since liver was energy-consuming compared to other organs, we applied a relatively high percentage of mitochondrial contents at 30%. After quality control and filtering out low-quality and doublet cells, we obtained a total of 105442 live single cells from adult mouse liver at all time points (Fig. 1B). Quality control was also performed for spatial transcriptomics resulting in a total of 25995 remaining spots with an average of 3764 spots per sample on tissue and of 3676 genes per spot. Histological analysis by 10X Loupe Browser revealed an average of six cells per spatial transcriptomic spot as well as well construction of fatty live model with universe lipid drops in hepatocytes (Fig. 1E, upper panel).

A well-integrated atlas of liver regeneration was constructed without batch effects at both normal and fatty status (Fig. S1A, C). The cells were categorized into Louvain clusters and annotated as 14 clearly differentiated major types referring to marker genes curated from the literature (Fig. 1B, Fig. S1B). The markers for each major type used for major type identification were summarized in Table S1 and visualized in dot plot format (Fig. 1C). Analysis of the major cell types showed that both types of parenchymal cell components, hepatocytes and biliary epithelial cells (BECs), were reduced in the fatty state, whereas some nonparenchymal cells, including Kupffer cells, endothelial cells, and hepatic stellate cells, showed a similar tendency to be reduced. Several studies have suggested that Kupffer cells and endothelial cells play a key role in liver regeneration (Fig. 1D). Temporal analysis of composition showed that in the fatty state, a sharp increase in neutrophils, monocytes, and macrophages at day 2 but decreased at the day 4 and day 6, indicating an early response to injury (Fig. S1D).

**Molecular niche and structural variation following liver resection**

To faithfully characterize liver zones in spatial distribution, we first inspected the classical zonation marker Sds for Zone 1, Igfbp2 for Zone 2 and Oat for Zone 3. In situ mapping of the zonation marker indicated a well applicable of Sds, Igfbp2 and Oat for both normal and fatty status after PHx (Fig. 1E-G). Next, an unsupervised clustering molecular niche-based approach was utilized. The rationale is that the molecular niches represent structural building blocks and specific functions that can be used for comparison between slides. After initial quality control, each slide was first checked and identified spatial high variable genes (hvgs). Then, we integrated spatial data and used a graph-based approach to cluster spots into seven molecular niches (Methods for details) without batch effects allowing for downstream molecular comparisons within the atlas across all slides (Fig. S1E-G). The compositions of each molecular niche were visualized in Fig. S1H.

To further characterize the features of each molecular niche, we performed differential analysis to identify the representative markers (Table S2). We found Niche 2 highly expressed periportal (Zone 1) genes such as Cyp2f2 and Sds, while Niche 1 and Niche 6 supported the pericentral genes expression consistent with previous studies such as Cyp2e1, Glul, Oat and Lect2 (Fig. 2A). The midlobular genes were enriched in Niche 3 and Niche 4 such as Igfbp2 indicating molecular niches could represent spatial architectures (Fig. 2A). The reported zonated markers were visualized for each Niche in volcano plot (Fig. 2C). However, there were no representative zonated markers for Niche 5 and Niche 7. We further projected the molecular niche onto H&E image and found bona fide maps between molecular niches and structures and showed representative images with clear zonation features. For example, the Normal_D2 slide showed a clear Zone 2 features with Niche 3 located between Niche 1 and Niche 2 (Fig. 2B). In the Fatty_D6 slide, the Zone 1 region showed portal vein was surrounded by Niche 2 and outside placed Niche 3 and Niche 1, while Zone 3 region turned on an opposite pattern with Niche 1 and Niche 6 located inner side, but Niche 3 and Niche 2 located outside (Fig. 2B). Moreover, we assign the zonal markers to all slides showing a distinguished zonation feature (Fig. 1E). Intriguingly, the Niche 5 and Niche 7 were located to exclusive inner or border regions of the tissue sections with partial overlap with liver tissues. Thus, the Niche 5 and Niche 7 were not used for niche-level specific analysis.

To further focus on the functional signaling pathways of tissue organization, we performed spatial-wide pathway comparisons on each spot per sample. Sharp increases were observed for Wnt pathway, a well-known positive liver regeneration related pathway, in normal mouse livers undergoing PHx (Fig. 2E, F). Despite the increasing phenomenon was also existed in fatty status, there were relative weakens in the activation degree of Wnt pathway (Fig. 2E, F). Since the liver was fine regulated by programmed activation and suppression, we also analyzed another suppression-related pathway TGF-beta signaling pathway (Fig. S2A, B). As the liver regeneration is more obvious in parenchymal hepatocytes, we further analyze the Wnt pathway and Cell cycle status of hepatocytes at the single-cell level. As a result, we found a relative delay of regeneration in fatty liver undergoing PHx. The Wnt pathway and proliferation activity peaked at day 2 in normal status, but the turning points were delayed and peaked at day 4 in fatty hepatocytes (Fig. 2D, G, Fig. S2C, D). Consistently, bulk RNA-seq data and western blot assays showed similar results that proliferation marker Pcna peaked at day 2 in normal status but delayed at day 4 in fatty status (Fig. 2H-I). In summary, these results indicated fatty liver applied to regeneration after PHx but the regeneration peak point was delayed at day 4.

**Interface hepatocytes up-regulate fetal programs in fatty status**

In the normal status, liver possesses great potential to regenerate after PHx mainly through the direct proliferation of remaining hepatocytes, while in fatty status the mechanism of regeneration remains unclear. To explore the mechanism of delayed regeneration in fatty status, we leveraged our single-cell RNA data integrating both hepatocytes and biliary duct cells (BECs). We noticed a subgroup residing between hepatocytes and BECs in UMAP plot (Fig. 3A). This subgroup highly expressed early hematopoietic markers including Vim and Cd44. Therefore, we termed these cells ‘‘interface hepatocytes’’. Functionally, in the fatty state, Vim+Cd44+ interface hepatocytes were more proliferative compared to other hepatocytes. However, some regenerative signals, such as Hippo and Wnt signaling activities, remained stronger in normal livers (Figure 3B, C). This suggests that the regenerative capacity of fatty liver is weak, and the Vim+Cd44+ interface hepatocytes may be a compensatory response under stress.

We used the smFISH method to validate the hematopoietic markers including Vim and Cd44 in the hepatocytes. Consistently, our results showed the signals of Vim and Cd44 were stronger at day 2 in fatty status compared to the normal status (Fig. 3D). A larger amount of Vim+Cd44+ interface hepatocytes existed at day 4 regarding day 2, which was the peak point of regeneration in fatty liver. While in the normal status, we noticed a weak signal of Vim and Cd44 at both day 2 and day 4. And we reprojected the Vim+Cd44+ interface hepatocytes in situ. A parallel result of our spatial transcriptional data showed a higher percentage of Vim+Cd44+ interface hepatocytes in both fatty day 2 and day 4 compared to the normal compartments (Fig. S3A-D). Similarly, the density of Vim+Cd44+ interface hepatocytes was more enriched on day 4 in fatty group and enriched on day 2 in normal group (Fig. S3A, D). Intriguingly, we noticed a spatial distribution discrepancy of Vim+Cd44+ interface hepatocytes. It was more abundant in Zone 3 in normal liver regeneration peak time (Day 2), while more abundant in Zone 1 in fatty liver regeneration peak time (Day 4, Fig. 3E). Normal livers regenerate mainly from residual hepatocytes, whereas the intermediate fetal state, i.e., Vim+Cd44+ interface hepatocytes, may be an essential source of regeneration in fatty livers.

**Spatiotemporal shifts of T cells in liver regeneration process**

Since immune responses during liver regeneration play an important role in promoting hepatocytes proliferation and regenerative response, we further characterized the immune features of other nonparenchymal cells including T cells, and myeloid cells. Similar to the processing of endothelial cells, we merged our single-cell data and clustered T cells into 12 subgroups based on the highly expressed genes and classic Cd4, Cd8 and NK cell markers (e.g. Ncr1; Fig. 4A, B, Table S1). Referring to the previous published work, we characterized the cytotoxicity, immunosuppression and inflammation ability of T subgroups (Fig. 4C). Here, we mainly focused on immune modulator Foxp3+Ctla4+ Cd4 T cells (namely Cd4+ Tregs) and inflammation modulator Ccl5+ Cd8 T cell. In normal status, both Cd4+ Tregs and Ccl5+ Cd8 T cells were less on day 2 (Fig. 4D). This helps to create a suitable immune environment for regeneration. When regeneration finished, the Cd4+ Tregs and Ccl5+ Cd8 T were accumulated to reverse the microenvironment and harness the regeneration procedure. However, in fatty status, the circumstances were reshaped with a persistent small amount of Cd4+ Tregs, early accumulated Ccl5+ Cd8 T at day 2 and decreased at day 4 (Fig. 4D). This may have led to an excessive immune response and injury that prevented early liver regeneration on day 2.

Next, we leveraged our Visium 10x spatial transcriptomics data to characterize these two subgroups across spatial and time ranges. With previously defined zonation region, our data has shown a zonation feature of Cd4+ Tregs and Ccl5+ Cd8 T. Compared to the other timepoint, the Cd4+ Tregs specifically re-located itself to Zone 3 to conduct the immunosuppressive function, while Ccl5+ Cd8 T seemed to be relative sparse over Zone 2 and Zone 3 to harness the regeneration at both regeneration turning point (Day 2 in normal status, Day 4 in fatty status, Fig. 4E, Fig. S3E-G). In short, these results showed the immune and inflammatory modulating functions of T cells as well as spatial location were pivotal for the essential performing regeneration function.

**Spatiotemporal shifts of endothelial cells function in liver regeneration process**

Liver endothelial cells are pivotal in regulating liver functions. However, differentiation in phenotype and function results from local microenvironmental signals based on anatomical location. Herein, we first used the single-cell data and removed the batch effects resulting in a total of six subgroups (Fig. S4A, B). By applying classical liver sinusoidal endothelial cells (LSCEs) marker Lyve1 and liver vascular endothelial cells (LVECs), we classified endothelial cells into types with anatomic features. We additionally apply classical zonation markers including pericentral Wnt2a and periportal Efnb2. The endothelial cells were well located with Kit+ LSCEs and Bmp4+ LVECs located in Zone 3, Mup11+ LSECs and Ccl21a+ LVECs located in Zone 1, while Ccr1+ LSECs and Mki67+ LSEC located between Zone 1 and Zone 3. Therefore, we assign the Ccr1+ LSECs and Mki67+ LSEC in Zone 2 region (Fig. S4C, D).

Next, we explore the function of endothelial cells among time and spatial dimensions. Among the six endothelial cell subgroups. Zone 3 endothelial cells (Kit+ LSCEs and Bmp4+ LVECs that highly expressed Wnt2) have been previously reported and validated to promote liver regeneration. Consistently, our data showed these two types were enhanced in regeneration pathways such as PI3K-Akt signaling and Hippo signaling pathway (Fig. S4E). However, the turning points were different among the two groups regarding immune regulations, inflammation, and cell proliferation (Fig. S4F). The immune modulating function was important for liver regeneration1. We noticed the immune suppressive function of Mup11+ LSCEs showed a similar trend to liver regeneration ability, indicating this LSCE might be another essential endothelial cell in liver regeneration (Fig. S4F).

**Dynamics of myeloid cells in liver regeneration process**

The myeloid cells are widely investigated in liver regeneration given their immuno-crosstalk feature. We used single-cell data and annotated 13 myeloid subgroups (Fig. S5A, B). The myeloid cells showed distinct distributions among timepoints in regeneration process. Spp1+ monocyte peaked in proportion at day 2 in both normal and fatty status, while Ccr7+ macrophage peaked at day 2 in normal status and at day 0 in fatty status. In this study, we focused on a subgroup termed Ace+ monocytes (Fig. S5C). This subgroup is more abundant at day 2 in normal status and at day 4 in fatty status, suggesting that it may have some contribution to liver regeneration. (Fig. S5D). We further used smFISH to validate the results. Similarly, the Ace+ monocytes were abundant in both liver regeneration peak timepoints. The normal liver presented more monocytes compared to the fatty liver on both day 2 and day 4 (Fig. S5F-I). We further explore the function of Ace+ monocytes showing this subtype is mainly involved in injury repair functions including focal adhesion, platelet activation, and phagocytosis to remove dead cells and avoid immune response (Fig. S5E).

**References**

1. Swiderska-Syn, M., Syn, W. K., Xie, G., et al. Myofibroblastic cells function as progenitors to regenerate murine livers after partial hepatectomy. *Gut.* 2014;*63*(8):1333-44.

2. Chen, Y., Meng, L., Xu, N., et al. Ten-eleven translocation-2-mediated macrophage activation promotes liver regeneration. *Cell Commun Signal.* 2024;*22*(1):95.

3. Chen, T., Oh, S., Gregory, S., Shen, X., Diehl, A. M. Single-cell omics analysis reveals functional diversification of hepatocytes during liver regeneration. *JCI Insight.* 2020;*5*(22).

4. Wesley, B. T., Ross, A. D. B., Muraro, D., et al. Single-cell atlas of human liver development reveals pathways directing hepatic cell fates. *Nat Cell Biol.* 2022;*24*(10):1487-1498.

5. Ghorbani, S., Jelinek, E., Jain, R., et al. Versican promotes T helper 17 cytotoxic inflammation and impedes oligodendrocyte precursor cell remyelination. *Nat Commun.* 2022;*13*(1):2445.

6. Chen, Y., Yang, S., Lovisa, S., et al. Type-I collagen produced by distinct fibroblast lineages reveals specific function during embryogenesis and Osteogenesis Imperfecta. *Nat Commun.* 2021;*12*(1):7199.

7. Kuppe, C., Ramirez Flores, R. O., Li, Z., et al. Spatial multi-omic map of human myocardial infarction. *Nature.* 2022;*608*(7924):766-777.

8. Ma, S., Sun, S., Geng, L., et al. Caloric Restriction Reprograms the Single-Cell Transcriptional Landscape of Rattus Norvegicus Aging. *Cell.* 2020;*180*(5):984-1001 e22.

9. Xue, R., Zhang, Q., Cao, Q., et al. Liver tumour immune microenvironment subtypes and neutrophil heterogeneity. *Nature.* 2022;*612*(7938):141-147.

10. Hildebrandt, F., Andersson, A., Saarenpaa, S., et al. Spatial Transcriptomics to define transcriptional patterns of zonation and structural components in the mouse liver. *Nat Commun.* 2021;*12*(1):7046.

11. Kleshchevnikov, V., Shmatko, A., Dann, E., et al. Cell2location maps fine-grained cell types in spatial transcriptomics. *Nat Biotechnol.* 2022;*40*(5):661-671.

12. Lange, M., Bergen, V., Klein, M., et al. CellRank for directed single-cell fate mapping. *Nat Methods.* 2022;*19*(2):159-170.

13. Chu, T., Wang, Z., Pe'er, D., Danko, C. G. Cell type and gene expression deconvolution with BayesPrism enables Bayesian integrative analysis across bulk and single-cell RNA sequencing in oncology. *Nat Cancer.* 2022;*3*(4):505-517.

14. Tang, F., Li, J., Qi, L., et al. A pan-cancer single-cell panorama of human natural killer cells. *Cell.* 2023;*186*(19):4235-4251 e20.

15. Tong, Y., Guo, D., Lin, S. H., et al. SUCLA2-coupled regulation of GLS succinylation and activity counteracts oxidative stress in tumor cells. *Mol Cell.* 2021;*81*(11):2303-2316 e8.
